# Association between matrix metalloproteinase-3 gene polymorphism and susceptibility to chronic periodontitis: A systematic review and meta-analysis

**DOI:** 10.5937/jomb0-49044

**Published:** 2024-09-06

**Authors:** Ankang Hu, Xin Wang, Lisi Ai, Kun Liu, Lingxue Kong

**Affiliations:** 1 Jinan Stomatological Hospital, Department of Endodontics, Jinan, China; 2 Jinan Stomatological Hospital, Department of Periodontics and Oral Mucosa, Jinan, China; 3 Jinan Stomatological Hospital, Department of Oral and Maxillofacial Surgery, Jinan, China

**Keywords:** matrix metalloproteinase-3, gene polymorphism, chronic periodontitis, meta-analysis, matriks metaloproteinaza-3, polimorfizam gena, hronični parodontitis, meta-analiza

## Abstract

**Background:**

This study aimed to explore the correlation between the Matrix Metalloproteinase-3 (MMP-3) 1171 5A/6A gene polymorphism and susceptibility to Chronic Periodontitis (CP).

**Methods:**

Following the PRISMA guidelines, a systematic search was conducted across four electronic databases (PubMed, Embase, Web of Science, and Cochrane Library) without any time or language limitations. The selection criteria included case-control studies examining the association between the MMP-3 gene polymorphism and CP. The data were independently extracted and cross-checked by two reviewers. The Newcastle-Ottawa Scale (NOS) was used to assess the quality of the studies. Statistical heterogeneity and publication bias were assessed.

**Results:**

Five studies, published between 2004 and 2019, met the inclusion criteria for the meta-analysis. No significant association was observed between MMP-3 gene polymorphism and CP susceptibility across all subjects in the four gene models. However, subgroup analysis revealed significant differences based on genotyping methods and smoking habits. Using PCR-RFLP genotyping method, the allele and additive models showed a positive correlation with the risk of CP (5A vs 6A, OR=1.12, 95%CI (1.02č 1.23); 5A5A vs 6A6A, OR=2.85, 95%CI (1.61č4.86)). In contrast, using Sanger sequencing method, the 5A mutation appeared to reduce CP susceptibility (5A vs 6A, OR=0.77, 95%CI (0.67č0.87); 5A5A vs 6A6A, OR= 0.20, 95%CI (0.09č0.42)). Moreover, smoking habits appeared to modulate the risk. Among smokers, the 5A mutation increased susceptibility to CP, while among nonsmokers it decreased.

**Conclusions:**

While no significant correlation was found in the overall population, the stratified analysis revealed nuanced relationships contingent on genotyping methods and smoking habits.

## Abstract (sr)

**Uvod:**

Ova studija je imala za cilj da istraži korelaciju između polimorfizma gena matriks metaloproteinaze-3 (MMP-3) 1171 5A/6A i osetljivosti na hronični parodontitis (CP).

**Metode:**

U skladu sa smernicama PRISMA, sprovedena je sistematska pretraga u četiri elektronske baze podataka (PubMed, Embase, Veb of Science i Cochrane Librari) bez ikakvih vremenskih ili jezičkih ograničenja. Kriterijumi za odabir uključivali su studije slučaja i kontrole koje su ispitivale povezanost polimorfizma gena MMP-3 i CP. Podatke su nezavisno izdvojila i unakrsno proverila dva recenzenta. Za procenu kvaliteta studija korišćena je Njukasl-Otava skala (NOS). Procenjena je statistička heterogenost i pristrasnost objavljivanja.

**Rezultati:**

Pet studija, objavljenih između 2004. i 2019. godine, ispunilo je kriterijume za uključivanje u meta-analizu. Nije primećena značajna povezanost između polimorfizma gena MMP-3 i osetljivosti na CP kod svih subjekata u četiri genska modela. Međutim, analiza podgrupa otkrila je značajne razlike na osnovu metoda genotipizacije i navika pušenja. Koristeći metodu PCR-RFLP genotipizacije, alelni i aditivni modeli su pokazali pozitivnu korelaciju sa rizikom od CP (5A naspram 6A, OR=1,12, 95% CI (1,02č1.23); 5A5A naspram 6A6A, OR=2,85, 95% CI (1,61č4,86)). Nasuprot tome, korišćenjem Sangerove metode sekvenciranja, činilo se da mutacija 5A smanjuje osetljivost na CP (5A naspram 6A, OR=0,77, 95% CI (0,67č0,87); 5A5A naspram 6A6A, OR=0,20, 95% CI (0,09č0,42)). Štaviše, činilo se da navike pušenja moduliraju rizik. Kod pušača mutacija 5A je povećala osetljivost na CP, dok je kod nepušača smanjena.

**Zaključak:**

Iako nije pronađena značajna korelacija u ukupnoj populaciji, stratifikovana analiza je otkrila nijansirane odnose zavisne od metoda genotipizacije i navika pušenja.

## Introduction

Chronic periodontitis (CP) is a globally prevalent and multifactorial inflammatory disease characterized by progressive destruction of the supporting tissues of the teeth, primarily driven by bacterial infection. This condition leads to gingival inflammation, alveolar bone loss, and if untreated, eventual tooth loss [1] [2]. It’s clear that periodontal bacteria serve as the initial factor in the pathogenesis of CP. However, recent studies have pointed out that the progression and severity of the disease largely hinge upon the host’s to these bacteria and their metabolic byproducts [3] [4]. The pathogenesis of CP entails an intricate interplay of host immune responses and the microbiota in the periodontal pocket, with a plethora of cytokines and inflammatory mediators participating in the complex network of periodontal tissue inflammation and immune response [5]. Within this network, Matrix Metalloproteinases (MMPs), particularly MMP-3, have been recognized to play a central role. MMPs comprise a broad family of zinc-dependent endopeptidases that govern (ECM) remodeling and degradation [6] [7]. These enzymes degrade several ECM components, including but not limited to collagen, elastin, gelatin, matrix glycoproteins, and proteoglycans.

MMP-3, also known as stromelysin-1, stands as a critical member of the MMP family due to its broad substrate specificity and its unique ability to activate other MMPs. Genetic alterations, such as single nucleotide polymorphisms (SNPs), can significantly influence MMP-3 transcription levels, protein production, and therefore, the overall functioning of the MMP-3 gene [8]. The MMP-3 gene, situated at chromosome 11q22.3, has an adenine nucleotide insertion at the 1171 position. This insertion leads to a promoter polymorphism of the MMP-3 gene, giving rise to two allelic variants – one with five adenine nucleotides (5A), and the other with six adenines (6A) [5]. This promoter gene polymorphism has been reported to affect the expression and regulation of the MMP-3 gene and has been proposed to associate with susceptibility to CP [9].

Current research concerning the association between MMP-3 gene polymorphism and CP susceptibility is sparse and often inconclusive. The objective of the present study is to perform a comprehensive systematic review and meta-analysis of the available literature to explore this association more precisely. In doing so, our intention is to enhance the current understanding of the genetic components involved in CP’s pathogenesis and their correlation with disease susceptibility. By scrutinizing the link between MMP-3 polymorphism and CP, this study endeavors to shed light on potential genetic markers for CP susceptibility, contributing to the predictive, preventive, and personalized medicine in periodontology.

## Materials and methods

### Search strategy

Throughout the systematic review procedure, we upheld compliance with the Preferred Reporting Items for Systematic Reviews and Meta-Analyses (PRISMA) guidelines [10]. Four electronic databases PubMed, Embase, Web of Science, and Cochrane Library were searched on May 9, 2023 and no time limitation was applied. Vocabulary and syntax were specifically adapted according to the database. The specific search terms of PubMed were: (»Matrix Metalloproteinase 3« (Mesh) OR »MMP-3« OR »matrix metalloproteinase-3«) AND (»Polymorphism, Genetic« (Mesh) OR »polymorphism«) AND (»Perio dontitis« (Mesh) OR »periodontitis« OR »Chronic Periodontitis« (Mesh) OR »chronic periodontitis«). There were no restrictions imposed on the language used. The reference lists of pertinent articles were manually scrutinized to identify any potential additional records.

### Inclusion criteria

The systematic review required that the studies included met specific criteria [11]: 1) Studies investigating the association between the MMP-3 1171 5A/6A gene polymorphism and susceptibility to periodontitis, which also must be case-control studies; 2) In the case group, patients meet the diagnostic criteria for CP. The control group consists of individuals with no periodontal inflammation and no systemic diseases; 3) The genotype experiment data for both groups are clear, complete, and obtainable, including Odds Ratio (OR) and corresponding 95% Confidence Interval (CI).

The exclusion criteria were as follows [11]: 1) Studies where the case group includes individuals with severe systemic diseases that might affect periodontal status; 2) Documents that lack comprehensive or unambiguous analytical data; 3) Case reports, commentaries, expert opinion, and narrative reviews.

### Data extraction

The literature screening and data extraction shall be carried out independently by two evaluators, and cross-checked, and if there are differences, the differences will be discussed and resolved. The data to be extracted included: first author’s name, year of publication, geographical region, disease/condition, number of cases, genotype of case group (5A5A, 5A6A, 6A6A), genotype of control group (5A5A, 5A6A, 6A6A), genotyping method, smoking habits. When there is no data of interest in the published report, we contact the investigators of the original study.

### Quality assessment

Two independent reviewers assessed the included studies quality using the Newcastle-Ottawa Scale (NOS), which comprises nine components distributed across three categories. These categories evaluate potential sources of bias, including selection, comparability, and outcome. Each study was then assigned a quality score ranging from 0 to 9. Studies scoring between 0–3 were categorized as low quality, those with a score of 4–6 were considered of medium quality, and those achieving a score of 7–9 were classified as high-quality studies. This structured quality assessment approach ensures a robust and consistent evaluation of the included studies.

### Statistical analyses

Chi-square statistics and the magnitude of I^2^ were utilized to gauge the degree of heterogeneityacross the studies. An absence of detected heterogeneity was suggested by an I^2^ value of 0%, while a value exceeding 50% signaled substantial heterogeneity. The symmetry of the funnel plot and Egger’s test were employed to inspect the potential for publication bias in the meta-analyses. In the event of an asymmetric funnel plot, we introduced assumed unreported negative studies to investigate whether publication bias had a significant effect on the impact estimates. For all statistical examinations, a two-tailed P-value of less than 0.05 was deemed statistically meaningful. The analysis of data was carried out using Stata version 17 (StataCorp, College Station, TX, USA).

## Results

### Search results and study selection

**Figure 1:**
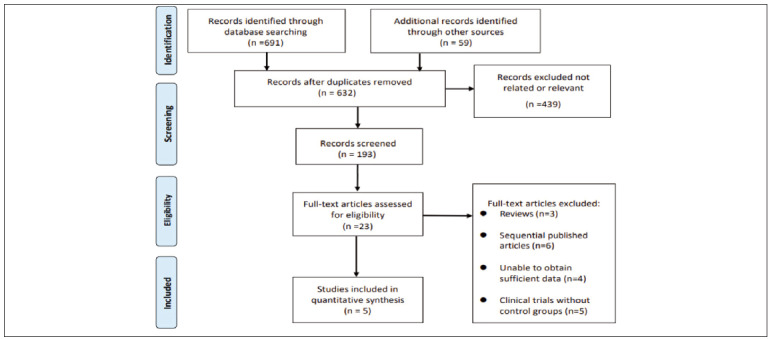
Selection process of included studies.

From the initial search of the electronic databases, 750 related literatures were initially found. After removing repetitive literatures, reading titles and abstracts, and screening strictly according to the inclusion and exclusion criteria, 23 related literatures were obtained, and 18 were excluded from further reading. Finally, 5 articles were included [5] [12] [13] [14] [15]. The literature screening process and results are shown in Figure 1.

### Study characteristics

The characteristics of studies included in this systematic review are presented in Table 1. The meta-analysis under discussion incorporates five studies published over a span of 15 years (from 2004 to2019) from various regions including Japan, Brazil, China, and India. Each study was centered on CP. The case group sizes varied across studies with the smallest group consisting of 114 individuals and the largest group containing 280 individuals. The control group sizes also differed significantly among the studies, ranging from 109 to 532 individuals. In terms of genotypes within the case groups, there were significant differences in the distribution of the 5A5A, 5A6A, and 6A6A genotypes. The studies utilized different genotyping methods; these include TaqMan PCR, PCR-RFLP, and Sanger Sequencing.

**Table 1:** Characteristics of studies included in the meta-analysis. SD, Standard deviation; CV, coefficient of variation

First<br>Author	Year	Country	Disease<br>Type	Case<br>Group Size	Control<br>Group Size	Case Group<br>Genotype	Control Group<br>Genotype	Genotyping<br>Method	Smoking<br>Status
Itagaki	2004	Japan	CP	205	142	5/58/142	4/38/100	TaqMan PCR	No
Astolfi	2006	Brazil	CP	114	109	19/52/19	8/70/25	PCR-RFLP	No
Lee	2011	China	CP	280	250	154/115/11	100/135/15	PCR-RFLP	No
Li	2012	China	CP	122	532	75/44/3	213/283/36	PCR-RFLP	Mixed
Majumder	2019	India	CP	157	200	72/56/29	134/56/10	Sanger<br>Sequencing	Mixed

### Results of quality assessment

We assessed the methodological quality of each RCT using the New Castle-Ottawa Scale (NOS). In general, two studies scored 8 points, and three studies scored 9 points. Blinding was not implemented in any of the studies, and there was a lack of indication of allocation concealment. There was no indication of funding biases in any of the studies. No studies were found to have incomplete outcome data, early stop-page bias, or baseline imbalances. Table 2 provides a summary of the potential risks associated with bias and their corresponding ratios.

**Table 2:** The quality assessment according to NOS of each cohort study. NOS: New Castle-Ottawa Scale

Study	Selection				Comparability	Outcome			Total<br>score
	Representativeness of<br>the exposed<br>cohort	Selection<br>of the non-exposed<br>cohort	Ascertainment<br>of exposure	Demonstration<br>that outcome	Comparability<br>of cohorts	Assessment<br>of outcome	Was follow up long <br>enough	Adequacy<br>of follow up of<br>cohorts	
Majumderet al. 2019 [5]	★	★	★	★	★★	★	★	★	9
Li et al.2012 [12]		★	★	★	★★	★	★	★	8
Lee et al.2011 [15]	★	★	★	★	★★	★	★	★	9
Astolfi et al.2006 [14]	★	★	★	★	★★	★		★	8
Itagaki et al.2004 [13]	★	★	★	★	★★	★	★	★	9

### Correlation between MMP-3 gene polymorphism and susceptibility to periodontitis

**Figure 2:**
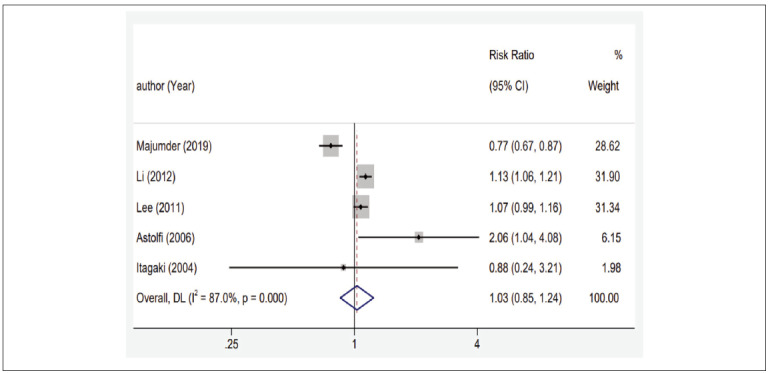
Forest plots of the association between MMP-3 1171 5A6A gene polymorphism and susceptibility to chronic periodontitis.

**Figure 3:**
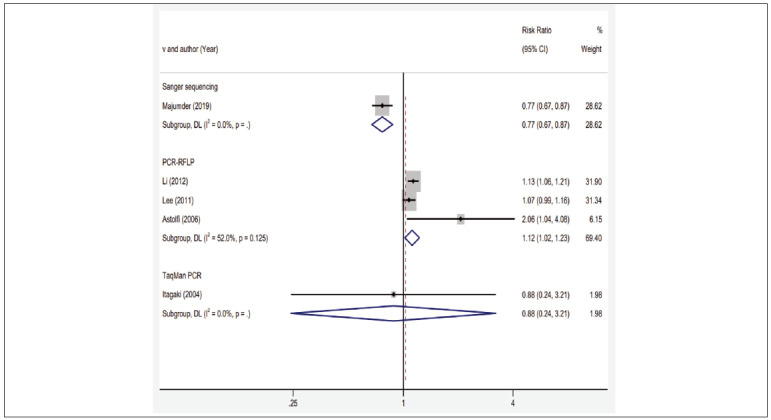
Subgroup analysis of the association between MMP-3 1171 5A6A gene polymorphism and susceptibility to chronic periodontitis.

No significant correlation was found between MMP-3 gene polymorphism and susceptibility to CP under four gene models among all the study subjects as shown in Figure 2. However, subgroup analysis based on gene analysis methods and smoking habits indicated a different trend as shown in Figure 3. In the subgroup analysis conducted using the PCR-RFLP genotyping method, the allele and additive model showed correlation with the risk of CP onset (5A vs 6A, OR=1.12, 95%CI (1.02~1.23); 5A5A vs 6A6A, OR=2.85, 95%CI (1.61~4.86)), suggesting that carrying the 5A mutation may increase the susceptibility to CP under the PCR-RFLP genotyping method. Conversely, under the Sanger sequencing genotyping method, all four gene model groups exhibited correlation with the risk of CP onset (5A vs 6A, OR=0.77, 95%CI (0.67~0.87); 5A5A vs 6A6A, OR=0.20, 95%CI (0.09~0.42); 5A5A vs 5A6A+6A6A, OR=0.44, 95%CI (0.28~0.67); 5A5A+5A6A vs 6A6A, OR=0.24, 95%CI (0.11~0.50)). This implies that carrying the 5A mutation might reduce the susceptibility to CP under the Sanger sequencing genotyping method as shown in Figure 3 and Table 3. Subgroup analysis based on smoking habits revealed a correlation between all gene models, except the dominant model, and the risk of CP onset (Smokers: 5A vs 6A, OR=1.34, 95%CI (1.08~1.64); 5A5A vs 6A6A, OR=2.00, 95%CI (1.17~3.55); 5A5A vs 5A6A+6A6A, OR=1.95, 95%CI (1.40~2.60). Non-smokers: 5A5A vs 5A6A+6A6A, OR=0.25, 95%CI (0.19~0.34)). This suggests that carrying the 5A mutation may increase the susceptibility to CP among smokers, but decrease it in non-smokers. For instance, consider the analysis for 5A5A vs 6A6A as shown in Table 3.

**Table 3:** Meta-analysis of the Association between MMP-3 1171 5A6A Gene Polymorphism and Susceptibility to Chronic Periodontitis.

Gene Model	Number of Studies	Susceptibility Analysis: OR	95%CI	P-value
5A vs 6A	5	1.03	(0.85~1.24)	0.70
PCR-RFLP	3	1.12	(1.02~1.23)	<0.001
Sanger Sequencing	1	0.77	(0.67~0.87)	<0.001
Taqman	1	0.88	(0.24~3.21)	0.86
Smokers	3	1.34	(1.08~1.64)	<0.001
Non-Smokers	2	0.93	(0.21~3.95)	0.76
5A5A vs 6A6A	5	1.36	(0.40~4.40)	0.63
PCR-RFLP	3	2.85	(1.61~4.86)	<0.001
Sanger Sequencing	1	0.20	(0.09~0.42)	<0.001
Taqman	1	0.92	(0.24~3.50)	0.68
Smokers	3	2.00	(1.17~3.55)	0.01
Non-Smokers	2	0.92	(0.05~20.00)	0.89
5A5A vs 5A6A+6A6A	5	0.78	(0.24~2.45)	0.66
PCR-RFLP	3	0.94	(0.12~7.00)	0.96
Sanger Sequencing	1	0.44	(0.28~0.67)	<0.001
Taqman	1	0.90	(0.25~3.20)	0.86
Smokers	3	1.95	(1.40~2.60)	<0.001
Non-Smokers	2	0.25	(0.19~0.34)	<0.001
5A5A+5A6A vs 6A6A	5	1.05	(0.51~2.08)	0.90
PCR-RFLP	3	1.66	(1.01~2.56)	0.06
Sanger Sequencing	1	0.24	(0.11~0.50)	<0.001
Taqman	1	1.11	(0.68~1.70)	0.56
Smokers	3	1.24	(0.85~1.70)	0.68
Non-Smokers	2	1.00	(0.75~1.33)	0.86

### Publication bias

**Figure 4:**
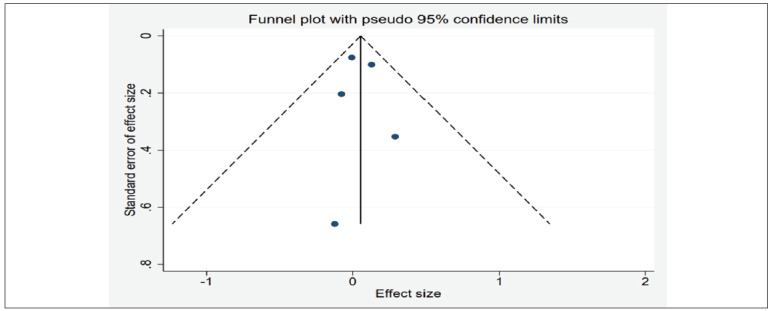
Funnel plot for publication bias in all included studies.

The funnel plots generated from the observed study exhibited symmetrical distribution, and no statistically significant evidence of publication bias was observed in the funnel plots (Figure 4).

## Discussion

The MMP-3 gene polymorphism has been a subject of interest due to its potential role in the pathogenesis of CP. Previous investigations, however, have revealed conflicting findings, underscoring the need for our meta-analysis. Our study demonstrated no significant association between MMP-3 1171 5A/6A polymorphism and overall CP susceptibility. However, in a stratified analysis based on genotyping methods and smoking habits, certain interactions emerged, illuminating the nuanced nature of this relationship. MMP-3 is pivotal in degrading extracellularmatrix components during inflammation, linking it to CP pathogenesis, marked by persistent inflammation and subsequent destruction of periodontal tissues. Hence, variations in the MMP-3 gene that could modulate its expression or activity might impact the inflammatory process and the susceptibility to CP. However, the precise mechanism through which MMP-3 gene polymorphism influences CP risk remains to be determined and is likely to be multifaceted.

Our findings resonate with the works of Li et al. [12] and Lee et al. [15], who observed an association between MMP-3 1171 polymorphism and CP susceptibility. This concurrence suggests that the 5A allele may reduce the risk of CP in the Chinese population. Astolfi et al. [14] also suggested that MMP-3 gene polymorphism is associated with periodontal tissue destruction in CP among Brazilians, indicating that the 5A allele could be a risk factor for CP development. Contrarily, the 6A allele has been associated with higher MMP-3 levels in patients with coronary heart disease and myocardial infarction [5], indicating the intricate relationship between gene polymorphisms,disease susceptibility, and the potential modulatory role of local and systemic conditions. The discrepancies found in various studies may be attributed to geographical factors, population migration, and genetic admixture, along with the close proximity of the MMP-1 and MMP-3 genes, which may give rise to linkage disequilibrium [16] [17].

In this meta-analysis, we did not observe a significant correlation between MMP-3 1171 5A6Agene polymorphism and the general population’s susceptibility to CP. However, subgroup analysis based on genotyping methods and smoking habits suggested that carrying the 5A mutation might increase susceptibility to CP in an additive model and among smokers, whereas it could decrease susceptibility among non-smokers. One important factor influencing our results is the presence of other polymorphisms in the vicinity, such as those in the MMP-1 gene located near MMP-3 on chromosome 11q22.3 [14] [16]. These neighboring polymorphisms might collectively impact the expression of MMP-3. A noteworthy point to consider is the complex regulatory mechanisms governing MMP-3 mRNA transcription [18]. The regulation is so intricate that the absence of a single MMP gene, such as MMP-2, MMP-3, MMP-7, MMP-8, MMP-11, or MMP-12, does not manifest as an observable disease phenotype in mice [19]. This suggests that the influence of a single MMP-3 SNP may be subtle or inconclusive in determining disease susceptibility or progression. Our systematic review and meta-analysis present a broad exploration of the association between MMP-3 gene polymorphism and CP susceptibility. However, it is essential to remember that periodontitis is a multifactorial disease, where the interplay between genes, environment, and lifestyle plays a vital role in disease pathogenesis. Furthermore, understanding the role of MMP-3 gene polymorphism in CP may have broader implications. For instance, this polymorphism has been linked to various forms of cancer, rheumatoid arthritis, and cardiovascular diseases. The further dissection of MMP-3’s role could thereby contribute to our understanding of the molecular mechanisms underpinning these diseases [20] [21] [22].

Despite these insights, this study is not without limitations. The lack of raw data from individual studies limited our ability to control for potential confounders, such as age, gender, and other risk factors. Also, the sample size and the number of included studies were relatively small, which may reduce the statistical power. Furthermore, publication bias can arise from various sources, including selective reporting and unpublished studies, which can significantly impact the overall results. Selective reporting can lead to an incomplete or biased representation of the true effects of an intervention or treatment. Unpublished studies, particularly those with negative or non-significant results, may not be readily available in the literature, leading to an overestimation of the treatment effect if only published studies are considered. Therefore, the results of the present research should be mindful of these biases.

In conclusion, while our meta-analysis marks a significant step in understanding the relationship between MMP-3 gene polymorphism and CP, further investigations are warranted. The study’s findings suggest that smoking habits may interact with the MMP-3 gene polymorphism to influence chronic periodontitis susceptibility. This highlights the complex interplay between genetic factors and environmental exposures in disease development, emphasizing the need for personalized approaches to prevention and treatment strategies for chronic periodontitis. The future studies should consider a comprehensive approach, examining the interplay between genetic, environmental, and lifestyle factors contributing to the risk of CP. Such research would not only enhance our understanding of CP’s pathogenesis but may also offer valuable insights for personalized prevention and treatment strategies.

## Conclusions

Our systematic review and meta-analysis present a comprehensive evaluation of the associationbetween MMP-3 1171 5A/6A gene polymorphism and susceptibility to CP. While no significant correlation was found in the overall population, the stratified analysis revealed nuanced relationships contingent on genotyping methods and smoking habits.

## Dodatak

### Conflict of interest statement

All the authors declare that they have no conflict of interest in this work.
